# On the earliest Acheulean in Britain: first dates and *in-situ* artefacts from the MIS 15 site of Fordwich (Kent, UK)

**DOI:** 10.1098/rsos.211904

**Published:** 2022-06-22

**Authors:** Alastair Key, Tobias Lauer, Matthew M. Skinner, Matthew Pope, David R. Bridgland, Laurie Noble, Tomos Proffitt

**Affiliations:** ^1^ Department of Archaeology, University of Cambridge, Downing Street, Cambridge CB2 3DZ, UK; ^2^ Department of Human Evolution, Max Planck Institute for Evolutionary Anthropology, Deutscher Platz 6, 04103 Leipzig, Germany; ^3^ Terrestrial Sedimentology, Department of Geosciences, Eberhard Karls Universität Tübingen, Schnarrenbergstr 94-96, 72074 Tübingen, Germany; ^4^ School of Anthropology and Conservation, University of Kent, Canterbury, Kent CT2 7NR, UK; ^5^ Institute of Archaeology, University College London, 31–34 Gordon Square, London WC1H 0PY, UK; ^6^ Department of Geography, Durham University, Lower Mountjoy, South Road, Durham DH1 3LE, UK; ^7^ Department of Archaeology, Classics and Egyptology, University of Liverpool, 12–14 Abercromby Square, Liverpool L69 7WZ, UK; ^8^ Technological Primates Research Group, Max Planck Institute for Evolutionary Anthropology, Deutscher Platz 6, 04103 Leipzig, Germany

**Keywords:** Acheulean, Middle Pleistocene, Lower Palaeolithic Britain, Quaternary gravel terrace, handaxe, small lithic technology

## Abstract

Northern Europe experienced cycles of hominin habitation and absence during the Middle Pleistocene. Fluvial gravel terrace sites in the east of Britain and north of France provide a majority of the data contributing to this understanding, mostly through the presence or absence of stone-tool artefacts. To date, however, relatively few sites have been radiometrically dated, and many have not been excavated in modern times, leading to an over-reliance on selectively sampled and poorly dated lithic assemblages. This includes Fordwich (Kent, UK), where over 330 bifaces were discovered through industrial quarrying in the 1920s. Here, we present the first excavation and dating of artefacts discovered *in situ* at Fordwich, alongside their technological analysis and relationship to those previously recovered. The site is demonstrated to retain deposits of Lower Palaeolithic artefacts, with 251 flakes, scrapers and cores identified to date. Infrared-radiofluorescence (IR-RF) dating of feldspar reveals 112 artefacts to have come from levels dating to at least 570 ± 36 to 513 ± 30 thousand years ago (ka) and are most plausibly assigned to an MIS 14 deposition, with artefacts produced during MIS 15 (approx. 560–620 ka). Indeed, these IR-RF samples provide minimum ages for artefacts. Combined with evidence from exposures linked to the original quarrying activities, a similar MIS 15 age is suggested for the more than 330 handaxe artefacts discovered in the 1920s. The remaining excavated artefacts come from levels dated to between 347 ± 22 and 385 ± 21 ka (MIS 10 or 11), with this later age interpreted to reflect post-MIS 14 deposition by substrate gullying and solifluction. These data demonstrate Fordwich to be one of the earliest Palaeolithic sites in northwestern Europe, and to retain the only large Acheulean handaxe assemblage directly dated to pre-MIS 13. Thus, Fordwich is determined to be a crucial piece of the pre-Anglian Palaeolithic puzzle in northern Europe.

## 1. Introduction

The archaeological site of Fordwich, located in northeast Kent (UK), has played a prominent role in the history of British Palaeolithic research. Indeed, it is among the best known of the island's Lower Palaeolithic sites following the recovery of over 330 bifaces during commercial quarrying in the 1920s [1,2]. The site has informed debate surrounding the Lower Palaeolithic of Britain ever since [3–10]. Fordwich's place among the Acheulean of Britain, Europe and the wider world is not, however, well described or understood; largely owing to an absence of absolute dating and no formal excavations having ever been undertaken. This has resulted in the site often (and understandably) being overlooked or only mentioned in passing (e.g. [10–16]).

Although thought to be ‘one of Britain's oldest sites’ [17, p. 8], previous estimates have only been able to place the gravel at Fordwich as an early pre-Anglian context dating from 700 000–500 000 years ago (ka) (MIS 17–13) [7,15,17,18]. These estimates are primarily based on the dating (absolute or otherwise) of lower terraces in the Stour Valley and surrounding area [5,7,17,19]. The ‘rough’ form of the bifaces is also argued to attest to an early European-Acheulean age ([3,4,7]; although see Ashmore [20] and White [6]). A pre-Anglian date would place Fordwich among the few known British Palaeolithic sites from this period. Indeed, while Barnham [21], Beeches Pit [22,23], Swanscombe [24] and others [25,26] present proof of hominins more regularly occupying Britain from MIS 13 onwards, evidence of hominins at, or prior to, 500 ka is rare ([10,11,25], albeit growing [27].

A collection of footprints at Happisburgh Site 3 (Norfolk) dated to greater than 800 ka currently represent the oldest evidence of hominins occupying Britain ([28]; although see [29]). Suggested to be made by *Homo antecessor*, these 152 impressions were found in sediment associated with a ‘small’ collection of 78 flakes and cores ([27,28,30], p. 32). Flake and core technology has also been reported from the 700 ka Bytham River estuarine deposits at Pakefield, Suffolk, though the assemblage from there is limited to 32 artefacts [25,31]. Davis *et al*. [10] report four flake artefacts from Fakenham Magna and Sapiston (Suffolk) that potentially date to MIS 19 and 17 (respectively), although the artefacts were not directly associated with the electron spin resonance (ESR) dated layers, and the authors stress the potential for overestimation of the age using ESR [32]. A single biface from Rampart Field (Suffolk) potentially dates to MIS 17 (680 ± 26 ka), although again, it was recovered from several layers above (i.e. post-dating) the dated sediment and an MIS 15 age for the biface is preferred by the authors [10,32]. Similarly, historically collected handaxe assemblages from Brandon Fields and Maidscross Hill on the Bytham River (Suffolk) are suggested to derive from MIS 15 [10], but these sites have not yet been radiometrically dated.

Warren Hill (Suffolk) displays a large (2000+), historically collected assemblage of taphonomically diverse bifaces from sands and gravels deposited during the Anglian glaciation [32]. The more heavily rolled and cruder bifaces from this collection are, however, argued to have been discarded by hominins during MIS 15 and to represent reworked materials from the older Timworth terrace [10]. The handaxes from the Slindon Formation at Boxgrove (West Sussex), exceptional in terms of their levels of refinement and preservation [33], are well accepted at MIS 13 (500 ka) and represent some of the earliest *in situ* Acheulean artefacts discovered in Britain. In addition, seven other British sites, all with evidence of handaxes, are known from roughly 500 ka. This includes Happisburgh Site 1 (Norfolk), High Lodge (Suffolk) and Waverley Wood (Warwickshire) ([10,27] and references therein).

Further evidence from secure pre-MIS 13 Acheulean sites is known from northern France. Moulin Quignon (Somme Valley) provides the earliest reliable evidence of the Acheulean in north-western Europe, with handaxes dating from as early as MIS 17 having been discovered [12,13,27,34]. The MIS 17 site of La Noira in central France supports such an early occurrence [35,36]. While there is ambiguity regarding La Noira's inclusion within north-western Europe [13,35], the site provides an important reference point for the more northerly record under consideration here. Abbeville (Somme Valley, France), with handaxes dating to 500–550 ka, provides further evidence of pre-Anglian (pre-Elsterian) Acheulean populations in northern France [12,34].

Together, these sites indicate that the north-western limit of Europe was occupied from as early as MIS 21 or 25 (depending on the date assigned to Happisburgh Site 3 [30]), and that hominin populations repeatedly occupied northern European locations prior to 500 ka. Indeed, Davis and Ashton [10,16] argue that six phases of pre-MIS 12 occupation can be seen in the British Lower Palaeolithic record. Based on the chronology of current sites, northern France may similarly have seen a ‘punctuated long chronology’ (cf*.* [14]) of hominin occupation prior to 500 ka characterized by absence and habitation aligned with glacial cycles, although recent research suggests at least some evidence of habitation during cold stages [13,34]. Yet, evidence for the Acheulean technocomplex (cf*.* [37,38]) in northwestern Europe prior to 500 ka is still not only rare but is temporally and geographically sporadic. Thus, questions remain about the frequency, duration, location and technological characterization of hominin occupations during this period.

Fordwich represents a crucial piece of this pre-Anglian Palaeolithic puzzle. Not only does it have a large and well-studied Acheulean handaxe collection, but it is located midway between the aforementioned French (Somme Valley) and British (Suffolk, Norfolk, West Sussex) sites in an area currently lacking any securely dated pre-Anglian artefacts. Moreover, its suggested 700–500 ka age potentially makes it one of the earliest Acheulean sites in northern Europe, and a possible contemporary of—and thus vitally needed technological, temporal and geographical comparison with—other important Acheulean sites, including Moulin Quignon, Abbeville and Warren Hill.

## 2. The site and artefacts of Fordwich

Despite being a well-known British Acheulean site, Fordwich has never undergone extensive formal excavation and the site itself has largely been overlooked for over 90 years. As a result, little is known about the age and origin of the well-documented handaxe artefacts now held at the British Museum. Since the 1930s, we can only find evidence of an additional 17 artefacts having been recovered from the site, with eight being found by John Wymer during a single test section dug in July 1977 [39], and a further nine flakes being discovered by Bridgland *et al*. [7] in two small section cuttings (see below).

**Figure 1.**
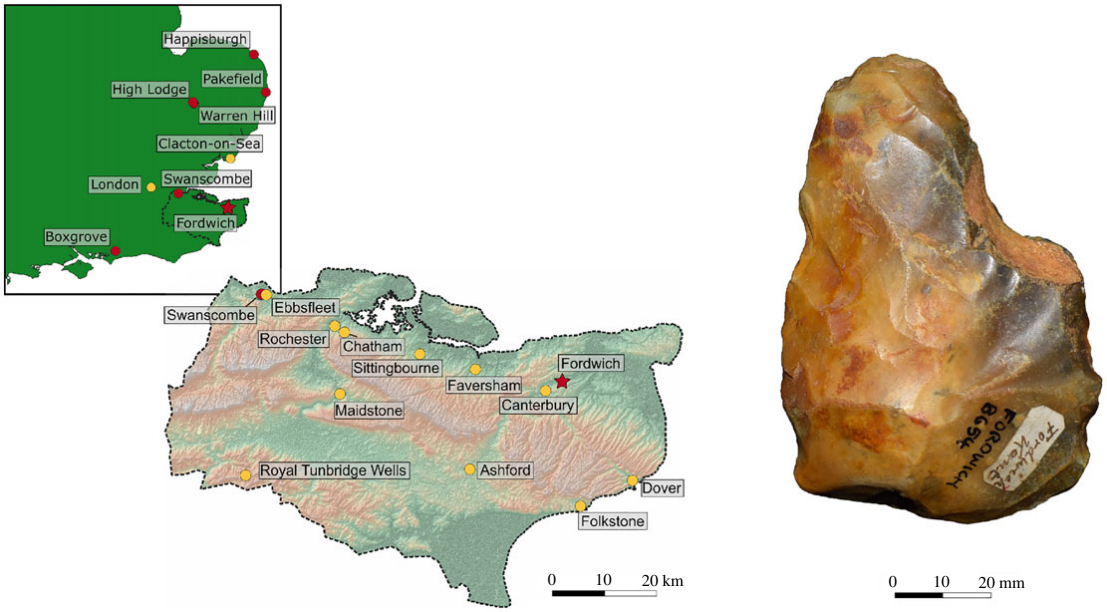
The location of Fordwich within Kent (UK) alongside an example of a handaxe discovered during quarrying in the 1920s. Red dots indicate Lower Palaeolithic sites; yellow dots indicate major towns or cities.

Fordwich Pit is a well-known Quaternary site in the Stour Valley (figure 1), with the valley displaying a rich clustering of Palaeolithic occurrences (see Bridgland *et al*. [7] and Wenban-Smith and Cuming [17] for wider discussion on the Stour Valley). The pit was formed through extensive gravel quarrying, with two sister pits having been excavated in the 1920s [1,2]. Here we report only from the west pit, which is more well-known, retains a greater number of artefacts in known collections, and is located further up the valley side away from the river Stour. Having been used for refuse disposal prior to the 1950s, and now used for agriculture, little evidence of the original quarry pit is retained. It is thought to have originally been roughly 350 × 150 m in size, with gravel having been worked to depths of between seven and twenty feet (roughly 2 to 6 m) [2]. The only known contemporary accounts of the quarry and its associated gravels are by Dewey & Smith [1] and Smith [2], although others have repeated these descriptions (e.g. [7,20]).

The terrace sequences in and around the Stour Valley have been covered in more detail, and in the vicinity of Canterbury and Sturry it is known to comprise three clearly demarcated terraces [7,40]. Fordwich Pit is located on the third terrace, *ca* 41–44 m OD (above ordnance datum (sea level)). The terrace sequence at Fordwich and Sturry has been suggested to indicate technological change through time [4], with the high-level gravels at Fordwich consisting predominantly of ‘crude, thick, narrow, pear-shape forms of limited technological sophistication… which have been argued to belong to a very early, archaic biface manufacturing tradition’ ([7], p. 51). Lower terraces at Sturry, on the opposite side of the Stour valley, display biface forms that are more traditionally considered ‘late Acheulean’ (this is suggested to be linked to raw material factors [6,7]), which are in turn overlain by Middle Palaeolithic technologies.

Texts contemporary with the recovery of the Fordwich Pit artefacts do not describe the site in detail or provide much information concerning their recovery, but it is known that the gravels are fluvial in origin and at their base are interstratified with sand [2,20]. According to Smith [2], this is followed by the main mass of gravel which displays little in the way of clear stratification, followed by a clear band of sand and one further thinner, upper layer of gravel, which is in turn covered by loam and soil. Although Smith [2] hypothesized that ‘Clactonian’ flake artefacts may have been concentrated towards the bottom of the gravel while the handaxes were more superior, there is no contemporary information on where in the Fordwich stratigraphy the handaxes were recovered. Bridgland *et al*. [7] note there to have been no clear artefact concentrations in the sections they excavated.

**Figure 2.**
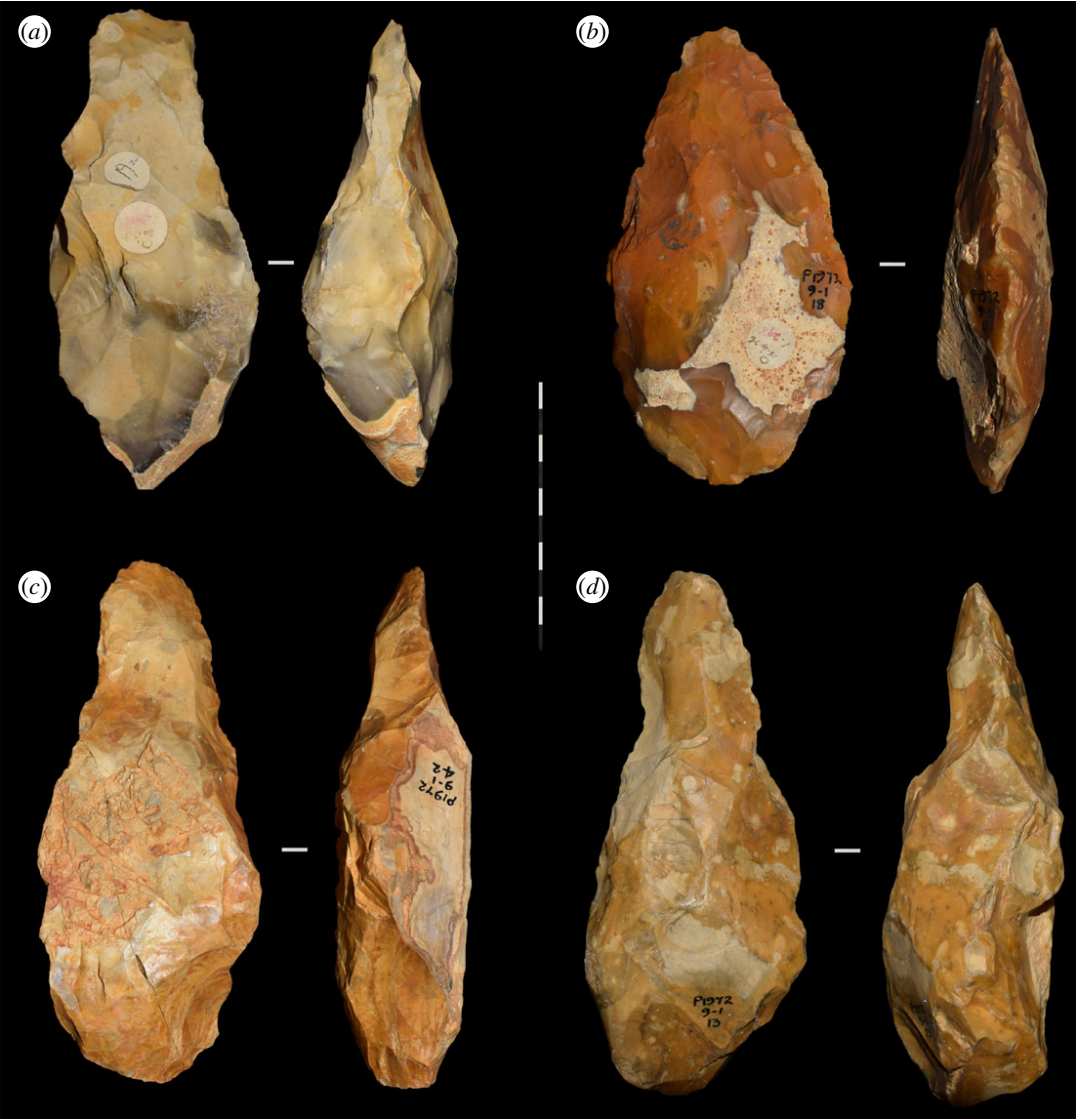
Four of the handaxes recovered from Fordwich Pit during the 1920s. Note that three display thick side profiles, elongated forms, and are relatively lightly worked (reduced) character (i.e. *a*, *c* and *d* could be considered ‘crude’). The fourth (*b*) is more heavily worked.

During the course of quarrying work, hundreds of handaxes and ‘a large number of amorphous flakes' were recovered from the pit by workmen and amateur archaeologists on an informal basis ([2], p. 169). Most of the handaxes are now stored at the British Museum, while smaller collections are held at regional museums, including Canterbury and Herne Bay. Ashmore [20] reports that, from the original more than 330, only 223 are currently accounted for, with 189 of these being stored at the British Museum. We can add an additional handaxe to this tally, having found a single example within the collections of Canterbury Museums Trust (figure 1). An unknown number of bifaces, flakes and cores entered private collections or were lost as part of the aggregate output. The handaxes are typically described to be narrow, thick and of ‘ovate tradition’ through to ‘pear-shaped’ [2,4,15], although there is notable variation and the presence of ficrons and other more pointed forms [20] (table 1). They are also noted to be ‘rough’ in their form, display irregular edges, and to retain substantial portions of cortex in many instances (although there are exceptions; figure 2). Little is known about the flake artefacts or the presence of retouched tools.

**Table 1.** Descriptive morphological data from 224 of the Acheulean handaxes recovered from Fordwich Pit during the 1920s, and now stored at the British Museum (*n* = 189), Herne Bay Seaside Museum (*n* = 34) and Canterbury Museums Trust (*n* = 1). Data for all artefacts are derived from Ashmore [20], except for the Canterbury Museums Trust handaxe. Note that some ratios were incorrectly calculated by Ashmore [20]; we provide corrected values.

	length (mm)	width (mm)	thickness (mm)	point of max. width from tip (mm)	elongation ratio (width/length)
mean	142.4	74.3	47.2	89.9	0.53
min	58.0	41.0	19.0	17.0	0.31
max	230.0	113.0	77.0	163.0	0.98
s.d.	29.5	118.0	125.0	23.6	0.09
CV (%)	20.7	15.9	26.4	26.2	17.6

## 3. Material and methods

### 3.1. New excavations at Fordwich Pit

**Figure 3.**
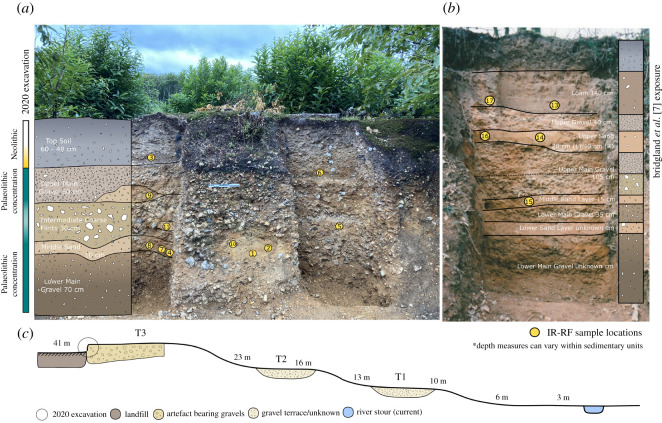
Trenches A (right (*a*)) and B (left (*a*)) following the 2020 fieldwork. Our trenches largely confirm Smith's [2] account of the stratigraphy within the main gravel. The upper main gravel returned a younger MIS 10/11 age compared with the lower main gravel dated to 570–513 ka. We interpret this younger age to reflect reworking of these upper gravels by slope processes, including gullying and solifluction, which accords with the site's position on the edge of the valley (i.e. the artefacts recovered from these layers still probably represent MIS 15 hominin activities). (*b*) depicts one of the two sections exposed by Bridgland *et al*. [7]. Note the loam and upper gravel, which is absent at Trench A and B, but accords with Smith's [2] description of the site. (*b*) is also at a different scale to (*a*), with the former's sediment being approximately three times as deep. In both (*a*) and (*b*), yellow circles indicate the location of the IR-RF samples detailed in table 2, while the numbers refer to the sample numbers in the same table. (*c*) shows a basic schematic depiction of the southern side of the Stour Valley at Fordwich. Terrace 3 (T3) bears the gravels containing the present artefacts and IR-RF samples at an altitude of 41 m OD. Note that the perspective in (*a*) makes the IR-RF samples in the middle non-excavated section appear lower relative to those at the back of the excavation. The gravels are also inclined to the right, such that layers are approximately 15 cm higher in Trench A than B. It is plausible that the lower sand layer in the 2020 excavation aligns with the lower sand layer in the 1998 exposure (this could not be verified at present due to sediment build-up at the base of the exposure preventing access for sampling). See electronic supplementary material, figures S1 and S2 for more detail.

Discoveries made during the first fieldwork season at Fordwich form the focus of the present article and represent the start of the first major archaeological work at the site in its 100-year history. Artefacts were recovered from two contexts. The majority come from two 1 × 1 m trenches dug into a portion of preserved gravel terrace on the edge of the quarry. Owing to the COVID-19 pandemic, work during 2020 was initially limited to these two small test trenches (‘A’ and ‘B’; figure 3). Other artefacts were recovered through ad hoc field walking, or from surface finds in the immediate vicinity of the two trenches.

The primary goal of the initial excavations was to confirm whether these sediments retained evidence for the presence of hominins in the form of lithic artefacts. To this end, three-dimensional point-plotting of the artefacts that were discovered was not undertaken. Instead, sediments were removed in 50 × 50 cm quarters with a spit depth of 10 cm. These were sieved through a 6 mm mesh screen with artefacts recovered being assigned to a particular 10 cm stratigraphic level, while a smaller number were recorded *in situ*. Where a spit cut across a sediment unit change, it was assigned to a layer as per table 3 depending on which layer contained the majority of the spit's depth (meaning that there is up to 4 cm of interchange between named layers in terms of artefact assignment).

### 3.2. Identification and analysis of lithic artefacts

Due to the fluvial deposition of the sediment [2,20] we anticipated there to be occasional challenges when identifying artefacts. Thus, a highly conservative approach to artefact identification was followed. All stone objects displaying features suggestive of anthropogenic modification were collected through sieving or *in situ* excavation. These objects were then subjected to analysis by three experienced Palaeolithic archaeologists (TP, MP and AK), two of whom have considerable experience identifying lithic artefacts from high-energy fluvial sediments, including in British Quaternary sequences. For an artefact to be considered for inclusion in subsequent technological analyses, agreement on its anthropogenic origin between all three analysts was required.

Artefact condition (degree of rolling) was subjectively assessed on a scale of 1–4, as is common in lithic archaeology (e.g. [10,41]). All artefacts were considered in natural lighting and made use of low-magnification optical aids. Once assessed, each artefact was grouped and compared with others of the same scale for verification. Methods to quantify differences in edge curvature (i.e. rolling) on lithic objects do exist [42,43], but for the present purposes these techniques were not deemed necessary. For reference, flakes ‘C’ and ‘D’ in figure 6 were scaled at level 4, flake ‘H’ was scaled at level 2, while the right flake in figure 8 was scaled at rounding level 1 (i.e. it was considered exceptionally fresh with little to no taphonomic damage).

### 3.3. Sample collection for infrared-radiofluorescence dating

Sixteen samples were taken for infrared-radiofluorescence (IR-RF) dating (figure 3). The samples were collected from freshly cleaned outcrops using light-tide steel tubes. The tubes were hammered into the wall and (after removing them) immediately sealed with light-tide tape and stored in black plastic bags.

Eleven were collected from the two excavated trenches (samples 1 to 11) while a further five were collected from the exposure investigated by Bridgland *et al*. [7] (samples 13 to 17; [note that sample 12 was purposefully missed). Within the trenches, samples were principally collected from sand lenses (samples 1, 2, 5, 6, 9, 10, 11) or the only visible band of sand (samples 4, 7, 8; figure 3). It was not possible to collect samples from the lowermost gravels as no sand lenses were exposed. Repeat samples were taken from several locations. Three samples could not be dated as there was only a very small amount of feldspar with the right grain-size (90–250 μm; samples 3, 6 and 8; table 2).

**Table 2.** IR-RF ages returned from the 16 sediment samples collected in 2020. Three age clusters are present in the sediment, with the oldest aligning to MIS 14 and the youngest aligning with MIS 10 or 11 and probably reflecting reworking of the upper main gravel (note the impact of error ranges on these associations). Sample nine from the upper main gravel (UMG) appears as an outlier relative to the sand lenses in the intermediate coarse flint (ICF) layer. This possible overestimation may be due to an incompletely bleached IR-RF signal caused by rapid transportation and burial of sediment. Ages could not be returned for three samples due to a lack of appropriately sized feldspar grains.

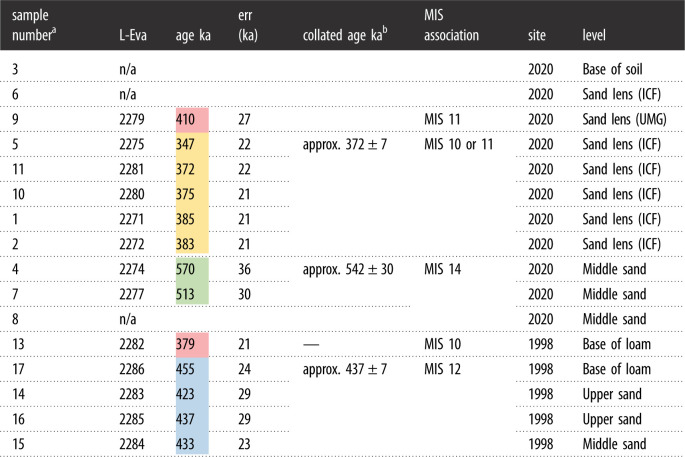

^a^No sample was assigned to number 12.

^b^Approximate mean age. The mean ages are based on the arithmetic mean of the ages belonging to one cluster. An age cluster was defined by a set of ages that are overlapping within the 1*σ* error range. The mean ages are presented with their standard error.

Given the close stratigraphic alignment of Bridgland *et al*.'s [7] exposure and Smith's [2] description of the gravels where the original handaxes were found, we took five samples from the former so that the artefacts discovered in the 1920s could be dated. Moreover, this allows us to place our current excavations within the wider framework of Smith's [2] description of the site. Permission was gained from Natural England prior to any samples being taken. Two samples were taken from the base of the loam above the thin uppermost gravel layer (samples 13 and 17); a further two were taken from the first (upper) sand layer in between two gravel layers (samples 14 and 16); and one final sample was taken from a lower sand layer located within the main gravel mass (sample 15; figure 3*b*).

### 3.4. Infrared-radiofluorescence dating methodology

To establish a chronological framework for the fluvial aggradation at Fordwich, we used IR-RF dating [44–46]. This method is a powerful tool for determination of the point at which feldspar grains were last exposed to sunlight, and hence to obtain information on the burial age of the deposits. The IR-RF signal saturates at doses mostly in between 1200 and 1500 Gy and thus allows the successful dating of Middle-Pleistocene sediments [47,48]. It is known that the IR-RF signal is more difficult to bleach if (for example) compared with the quartz optically stimulated luminescence (OSL) or infrared stimulated luminescence (IRSL) signals measured at lower temperatures (e.g. [44,49]). This makes the application of IR-RF challenging for young Holocene and mid-to-late Weichselian samples. Nevertheless, dose residuals should play only a minor role for Middle Pleistocene samples. Since Fordwich represents a fluvial aggradation of previously disposed stone tools the method will deliver the minimum age of the artefacts. Artefacts displaying considerable edge abrasion and rolling are likely to be older than the IR-RF dates indicate.

Sample preparation was conducted under subdued red light in the luminescence and infrared radiofluorescence laboratory at the Department of Human Evolution, Max Planck Institute for Evolutionary Anthropology (Leipzig, Germany). First, the outer 2 cm of sediment in the cylinders was removed to avoid any light-contamination. Subsequent treatment to separate the coarse K-feldspar grains included (as is standard) sieving and chemical treatment with 15% HCl, followed by the destruction of organic matter using 30% H_2_O_2_. The mineral-separation was done using sodium polytungstade. A density of 2.58 and 2.53 g cm^−3^ was used to separate the K-feldspar rich fraction. For De-measurements either the 90–250 µm or the 90–125 µm fraction was used. The wide grain size range (90–250 µm) was needed for most samples with respect to the limited amount of available coarse grain K-feldspar. All De-measurements were carried out on a Lexsyg research system with a freshly calibrated 90Sr/90Y beta ring source with a dose rate of approximately 0.51 Gy s^−1^.

The IR-RF signal was detected through a Hamamatsu PM tube and the signal was filtered through a Chroma D850/40 interference filter. The IRSAR protocol [50] was used for all equivalent dose (De) measurements and 3–13 aliquots of 5 mm diameter were measured per sample, depending on the available amount of coarse grain K-feldspar.

**Figure 4.**
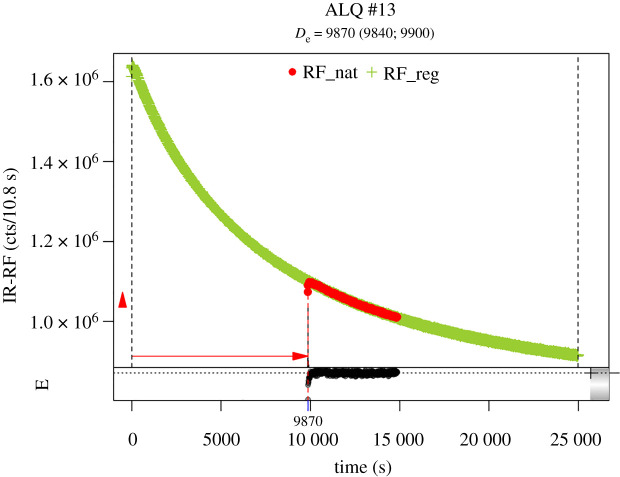
IR-RF curves obtained from sample L-Eva 2286. The red signal marks the natural IR-RF signal, measured for 5000s at 70°C. The green signal shows the regenerated IR-RF curve, that was detected during beta-irradiation after the aliquot was bleached. During the prior bleaching procedure, the IR-RF-related traps were emptied, and hence, the signal intensity was raised to its maximum level (high trapping probability per time).

**Figure 5.**
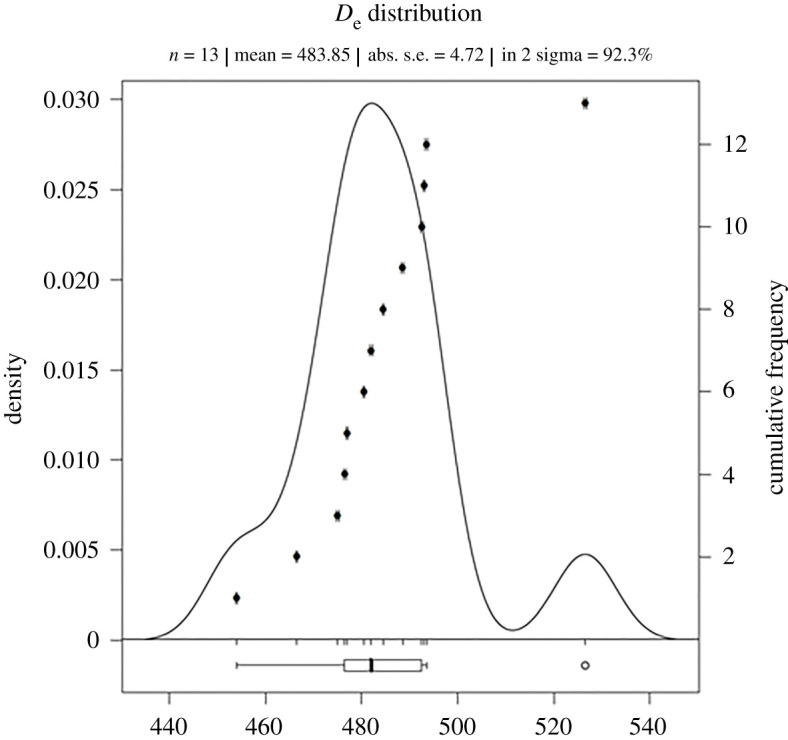
IR-RF De-distribution obtained from sample L-Eva 2286.

For data analysis the R software package (v. 3.5.1) was used including the R-luminescence package [51]. The age calculation was done with the Adele software (ADEL v. 2107). The mean De-value with its standard error was used for age evaluation respectively. Figure 4 shows representative IR-RF curves (natural and regenerated signal) from sample L-Eva 2286 and figure 5 shows the corresponding De-distribution of that sample.

The concentrations of U, Th and ^40^K were measured on the dried (bulk) sample material using low-level (high-resolution) gamma-ray spectrometry (N-type detector) at the VKTA (Felsenkeller laboratory) in Dresden, Germany. The specific activity of ^238^U was determined based on the activity of ^234^Th. ^214^Bi and ^214^Pb were used for ^226^Rn, ^228^Ac for ^228^Ra, and ^212^Pb and ^208^TI to get the activity of ^228^Th. The specific activity for ^232^Th is based on the weighted mean of the values for ^228^Ra and ^228^Th assuming an equilibrium for the ^232^Th decay-chain. To account for the cosmic dose rate contribution, the height a.s.l., geographical coordinates as well as the sampling depth below surface were considered. The evaluation of all dose rate components was done with the Adele software package (v. 2017) and the details on calculation can be found in Degering & Degering [52]. A value of 0.067 ± 0.012 [53] was used and the internal potassium content was estimated to be at 12.5 ± 0.5% [54]. The dosimetry data can be found in electronic supplementary material, table S1.

## 4. Results

### 4.1. Infrared-radiofluorescence results

The IR-RF ages and their 1 sigma error outlined in table 2 show that there are three age clusters with estimates that overlap in their error range. Two clusters, one at approximately 372 ka and another at approximately 542 ka, are located in our recent 2020 excavation, while the third approximately 437 ka date cluster is from the 1998 exposure investigated by Bridgland *et al*. [7] (figure 3*b*). These multiple clusters point to several periods of fluvial aggradation and/or reworking of the deposits, probably interrupted by pauses in fluvial activity or incision, followed by later reworking of the uppermost sediments by other processes, probably related to the valley-side slope (electronic supplementary material, figures S1 and S2).

The 347 to 385 ka ages for the upper main gravel and intermediate coarse flints at our excavations correlate with MIS 10/11. The lower main gravel broadly correlates with MIS 14. We interpret the younger age for the upper part of the sequence to be the result of later reworking of the uppermost part of the fluvial aggradation, as already described. Fluvial deposition at Fordwich is interpreted as having occurred during cold climatic periods with high-energy fluvial activity, potentially in a braided river system. Indeed, braided gravel-bed rivers point to periglacial climates in NW Europe, as does evidence of substantial gravel supply and deposition [24,55–57]. In combination with the abraded nature of many of the lithic tools we interpret the artefacts as having most likely been discarded by hominins during MIS 15. Note that MIS stage interpretations use central values and occasionally move one MIS stage up or down when more extreme error range values are considered.

The Bridgland *et al*. [7] exposure returned ages of between 379 ± 21 and 455 ± 24 ka for the upper sand layer and loam, and a date of 433 ± 23 ka for the middle sand layer; stratigraphic levels that are not present at our recent excavations. Caution should be applied to the age interpretation of the middle sand layer due to the lack of repeat sampling.

### 4.2. Archaeological units and stratigraphy

Two clear stratigraphic levels were present in each trench. The upper level is composed of topsoil intermixed with fine sand and flint gravel. There is a slight incline to the surface of the next (lower) level, which comprises dense gravel, running from trench ‘B’ to ‘A’ (left to right in figure 3) such that the first ‘soil’ section is 60 cm deep (Trench B) and the second is 48 cm deep (Trench A). This reflects an incline observed in the underlying sand (Lambeth Group sands) which affects the profile of all layer comparisons between the two trenches (i.e. levels in A are higher than in B) (electronic supplementary material, figure S1). Toward the bottom of the soil layer a thin band of loose gravel retains high levels of organic matter. Below this is the upper limit of the main gravel mass (i.e. the first geological unit), which continues for a further 190 cm.

We found no evidence of Smith's [2] clear band of sand above the main mass of gravel, nor the upper thinner layer of gravel and loam (figure 3). We are confident that this is not due to past quarrying as the presence of *in situ* Neolithic remains immediately superior to the excavated gravels demonstrates it to be undisturbed in recent times (see below). Moreover, it matches Smith's [2] description of an incline in the depth of the gravel from east to west. The only other remaining quarry exposures at Fordwich Pit do, however, closely match the description by Smith [2] in all ways (figure 3). These were the exposures investigated by Bridgland *et al*. [7] and clearly demonstrate the soil and loam overlying an upper, thinner band of gravel, which itself is overlying a sand layer. Beneath is the main gravel mass which becomes interstratified with sand toward its base.

The upper limits of the sediment at the 1998 exposure and 2020 excavation are broadly level, meaning that the stratigraphic units in the former are considerably deeper (both altitude and unit thickness) than in the latter (measured to the Lambeth Group sands) (electronic supplementary material, figure S2). Again, this is in line with the gravel depth (thickness) increasing toward the west of the pit as the base declines in line with the underlying slope. This means that the gravel at our slightly more easterly 2020 excavation rests on higher Lambeth Sands than the 1998 exposure. Notably, both the 1998 exposure and our new excavations are toward the extreme west of the pit where a letter contemporary with the original quarrying activity suggests most handaxe artefacts were found [4]. Our excavations are approximately 100 m north and approximately 30 m east of the Bridgland *et al*. [7] exposures, and closer to the edge of the valley. The Bridgland *et al*. [7] exposures are further from the valley edge and are positioned immediately before (i.e. marginally easterly to) a small fluvial gulley that demarks the edge of the pit. We interpret the absence of the loam, upper gravel layer and sand band at our 2020 excavation as little other than the result of lateral facies variation within the terrace sediment body. Indeed, the MIS 12 sediments at the Bridgland *et al*. [7] exposure probably result from later fluvial activity aggregating gravel above portions of the MIS 14 sediments (electronic supplementary material, figure S2).

At a depth of 50 to 80 cm from the surface of the gravel, flint sizes become noticeably larger in both excavated trenches, regularly reaching 10–15 cm in maximum diameter. Several sand lenses were visible within or just above this approximately 30 cm deep intermediate coarse flint layer (figure 3*a*). We term the finer gravels above this intermediate coarse layer the *upper main gravel*, while the finer flints below it are termed the *lower main gravel*. Clast size varies in these gravels, but flints are typically 1 to 7 cm in diameter and are supported in a cemented matrix of fine-grained clasts containing iron (as is typical for gravels of this age; electronic supplementary material, figure S6). In Trenches A and B, the intermediate coarse flint layer is underlain by an approximately 15 cm thick band of sediment with a high sand content. This could potentially align with the lower sand layer from the original Smith [2] description and later Bridgland *et al*. [7] exposures (figure 3*b*; electronic supplementary material, figures S1 and S2). Although the substantial (greater than 100 m) distance between the locations introduces some uncertainty. Despite only being approximately 20 m apart, there is variation in the sand and loam layers between the two 1998 exposures (figure 3; electronic supplementary material, figure S5). Such lateral variation is entirely to be expected in a fluvial depositional context; especially one interpreted to be part of a braided river system. The lack of clays and silts in the excavated gravels, along with evidence of bedding structures, supports our interpretation of a fluvial origin for these sediments (i.e. flowing water) as opposed to other process (e.g. solifluction). Variable clast sizes and the presence of a high sand-content layer point to diversity in fluvial energy through the sequence.

Roughly 145 cm from the top of the gravel in Trenches A and B sediment was noticeably looser with a higher sand content. Beneath this approximately 15 cm of looser, sandier gravel there was a continuation of the tightly packed gravel for a further 70 cm (electronic supplementary material, figure S1). Again, this matches the lower stratigraphy described by Smith [2] and exposed by Bridgland *et al*. [7]. With the exclusion of the loam and upper gravel/sand layer, the gravels at our recent excavations therefore broadly match Smith's [2] description of the main gravel mass from which the Fordwich handaxes were most likely recovered. Future work is planned to gain additional insights into the complex fluvial architecture of the site. This will provide a better understanding of the distinct MIS 14 and MIS 12 periods of fluvial aggradation, along with the erosion and/or reworking processes. Further IR-RF samples will be collected to aid this process.

### 4.3. Lower Palaeolithic artefacts

Lithic artefacts were recovered from both excavated trenches. Many had experienced taphonomic damage due to fluvial activities, being rolled and/or fragmented, while some retained very fresh edges indicative of little post-depositional modification. Using the techniques outlined in §3.2, a total of 251 artefacts were identified, comprising roughly 20% of the collected lithic material from the excavation. Artefacts were found throughout the excavated gravel, although two clear concentrations were present (table 3). The uppermost 50 cm of gravel (the upper main gravel) contained 113 (45.0%) artefacts, with the greatest concentration being 40–50 cm from the upper limit of the gravel (*n* = 41, 16.3%). The intermediate large flint layer at a depth of 50–80 cm contained fewer artefacts (*n* = 26, 10.4%). It is notable that Trench B had no lithic artefacts between 60 and 80 cm. The lower main gravel (incl. sand layer) contained 112 artefacts (44.6), with a concentration located 110–120 cm from the gravel's upper limit (*n* = 31, 12.4%).

**Table 3.** Absolute and relative frequencies of artefact concentrations throughout the excavated gravel sequence. Data are from both trenches combined, as measured from the surface of the main gravel mass. Note that the associated sediment layers are here for reference and do not perfectly align with the depth increments.

depth (cm)	no. artefacts	%	associated layer
0–10	22	8.76	upper main gravel
10–20	10	3.98	
20–30	21	8.37	
30–40	19	7.57	
40–50	41	16.33	
50–60	17	6.77	intermediate coarse flint
60–70	5	1.99	
70–80	4	1.59	
80–90	10	3.98	sand layer
90–100	14	5.58	lower main gravel
100–110	16	6.37	
110–120	31	12.35	
120–130	19	7.57	
130–140	16	6.37	
140–150	6	2.39	
total	251	100	

#### 4.3.1. Detached pieces

**Figure 6.**
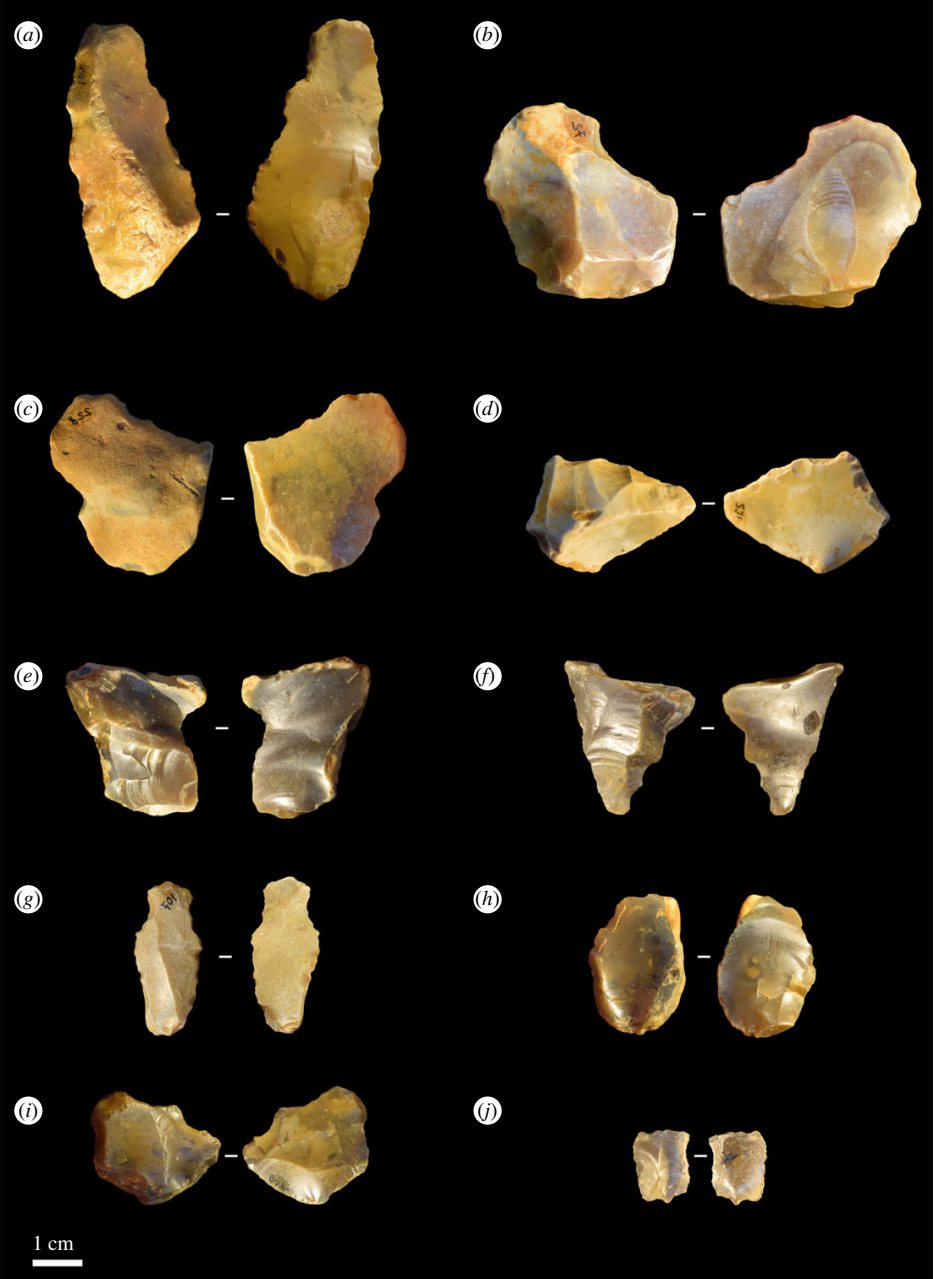
Flake artefacts from the 2020 excavations at Fordwich with varying levels of taphonomic damage. For example, flakes (*c*) and (*d*) were scaled at rounding level 4 and flake (*h*) was scaled at level 2.

The majority of the excavated lithic assemblage comprises detached products. A total of 238 flakes and flake fragments were identified within the excavated gravel, which represents 94.8% of the whole assemblage (figure 6). All were made of flint and displayed no evidence of thermic alteration. Most displayed some degree of patination (*n* = 213, 89.5%), although its extent varied between flakes. ‘Roundness’ and edge damage levels were also variable within the flake assemblage, with 159 (66.8%) displaying some damage along their edges and 175 (73.5%) displaying a degree of rounding (i.e. displaying rounding of ‘2’ or above on a scale of 1–4). A few were exceptionally well preserved and had probably experienced little to no disturbance after discard (‘scale 1’; *n* = 63, 26.5%). Most displayed light to moderate evidence of disturbance (‘scale 2’; *n* = 165, 69.3%), while a small number were more heavily rolled (‘scale 3’ or ‘4’; *n* = 10, 4.2%), and 65 displayed evidence of being snapped/fragmented (27.3%). Complete flakes ranged in size between 9.4 and 56.8 mm in length, 7.0–53.1 mm in width, 1.3–18.6 mm in thickness and 0.2–40.2 g in weight (table 4 and figure 6).

**Table 4.** Descriptive morphological data for the flake artefacts described here (*n* = 238).

	length (mm)	width (mm)	thickness (mm)	weight (g)
mean	22.5	19.6	5.5	3.7
min	9.4	7.0	1.3	0.2
max	56.8	53.1	18.6	40.2
standard deviation	7.9	7.6	2.8	5.2
coefficient of variation (%)	35.2	38.8	51.5	142.4

Seventy-two (30.3%) flakes possessed cortical striking platforms and 121 (50.8%) retained some evidence of dorsal cortex. Flakes displaying non-cortical platforms total 132 (55.5%), while those with no cortex on their dorsal surface total 117 (49.2%). Of those with non-cortical striking platforms, 53 (22.3%) retained evidence of platform preparation through faceting (a limited number with multiple facets appear typical of biface production, many are not clear), 36 (15.1%) had no visible evidence of preparation, and this was not clear for a further 43 (18.1%). Of those displaying evidence of previous flake removals on their dorsal surface (*n* = 156, 65.5%), an average of 2.1 removals were recorded. A few flakes displayed scars suggestive of secondary flaking (i.e. being flake cores; figure 6*e*), but fluvial processes could equally be responsible.

Multiple hinge-and-step terminations were present within the assemblage (41 and 4, respectively, 18.9%), alongside 10 plunging flakes where the termination had removed the basal portion of the core. Some 112 (47%) flakes displayed feather terminations while 72 (30.3%) were not clear. Of those displaying complete bulbs of percussion (*n* = 186, 78.2%), 64 (26.9%) were marked while 104 (43.7%) were more diffuse.

#### 4.3.2. Cores

**Figure 7.**
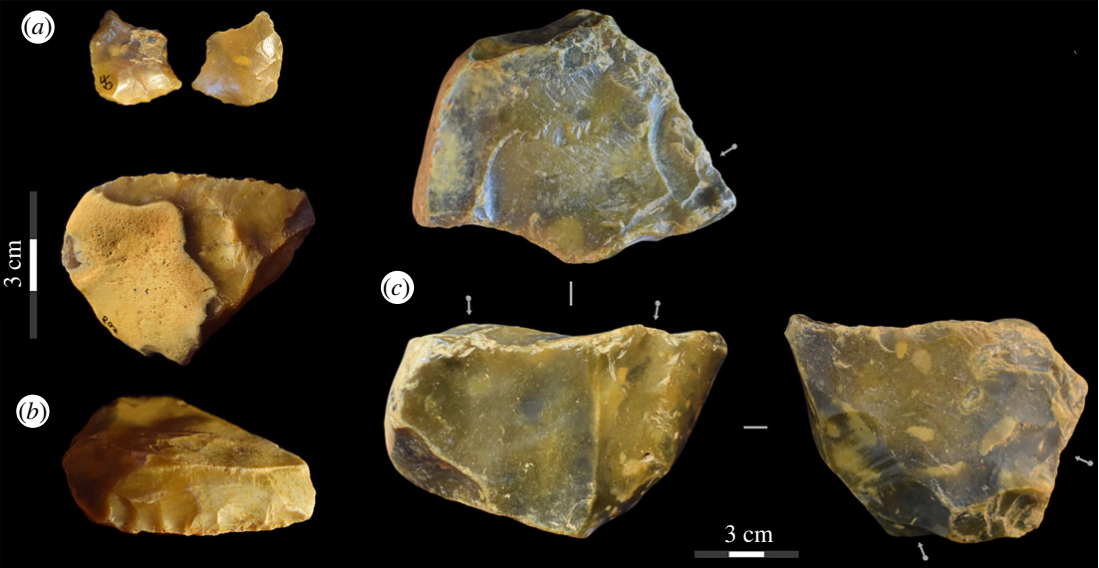
Examples of retouched pieces and a core from Fordwich Pit. The double pointed retouched implement is in the top left (*a*) while the largest of the scrapers can be seen in the bottom left (*b*). The largest core is on the right (*c*). See the electronic supplementary material, information for three-dimensional models of the core and double-pointed implement.

A total of four flint cores were identified (figure 7; electronic supplementary material, figure S3; Information 1). Three were small in maximum dimensions, measuring between 28.2 and 11.3 mm, and weighing 19.1–5.1 g. Two of these cores displayed cortex covering approximately 25% of their surface, while the other was non-cortical. Platform preparation was minimal, and no small cores possessed faceted platforms. No knapping accidents were present. Two of the small cores displayed four flake scars, while one appeared more highly exploited and had eight visible removals (although see discussion regarding its designation as anthropogenic; electronic supplementary material, figure S3). One small core is highly rolled, obfuscating the flake scars. The larger core weighed 284 g, had a maximum dimension of 84.5 mm, was exploited multifacially, displayed one unifaceted platform, displayed multiple misdirected impact points and two hinge fractures, and had seven visible flake scars (figure 7).

#### 4.3.3. Retouched pieces

A small number of potential retouched artefacts were identified. These included three scrapers, one double-pointed implement, and two notched flakes (figure 7), while a further flake displayed evidence of continuous retouch on a portion of its edge. The scrapers were fragmented, but the portions recovered were between 34 and 50 mm in length. The double-pointed implement is only 19 mm in length but displays clear direct, abrupt continuous retouch to produce two symmetrical points (figure 7; electronic supplementary material, Information S4). The scrapers displayed direct continuous edge modification (figure 7). All were made from flint and displayed light taphonomic damage, which was distinct from the deep, continuous removals indicative of intentional retouch. The recovery of these implements from fluvial deposits must, however, be stressed; non-anthropogenic flaking processes potentially contributed to the observed edge modification in some instances.

#### 4.3.4. Excavated neolithic artefacts

Immediately above the excavated gravel mass was a small collection of Neolithic artefacts, supporting our assertion that the intermediate sand layer and upper gravels found elsewhere at Fordwich (and described by Smith [2]) have not been removed through industrial quarrying processes. These finds are not the focus of the present article and will be described in future work, but include ceramics, flint blades, debitage and a hammerstone.

#### 4.3.5. Surface-discovered lithic artefacts

Twenty-two lithic artefacts were found through ad hoc field walking and in the immediate vicinity of the excavated trenches. Surface artefacts do not appear to be in particularly dense concentrations, but to what extent this has been influenced by the prior removal of artefacts as aggregate or collection we do not know. All are flakes, and a combination of Lower Palaeolithic and more recent periods of occupation are represented. This is in keeping with elsewhere in the Stour Valley.

## 5. Consistency with previous artefacts recovered at Fordwich Pit

Our 2020 excavations support Smith's [2] assertion that a ‘large number of amorphous flakes' are present at Fordwich. Indeed, in approximately 4 m^3^ of excavated gravels 238 morphologically diverse (shape and size) flake artefacts were discovered alongside 13 small retouched and core pieces. While similar concentrations of tools cannot automatically be assumed elsewhere at the site, it is reasonable to assume that several thousand of these small artefacts made their way into the aggregate output during the early twentieth century and were not retained alongside the handaxes. Given the limited number of flake artefacts from Fordwich retained in modern collections, there is clear evidence of artefact selection bias at this site during the first half of the twentieth century. A letter contemporary with the original quarrying at Fordwich suggests that most artefacts were found in the extreme west of the pit [4]. We cannot speak to this point in detail, but our trenches were located to the northwest of the pit and clearly retain strong evidence of flint artefacts.

We found evidence of artefact-based separation between upper and lower elements within the main gravel mass, but there were no clear stratigraphic trends in terms of rolling or pseudo retouch, with ‘fresh’ flakes being found throughout. There is a tendency for the most heavily rolled artefacts (those graded as ‘3’ or ‘4’) to be lower in the sequence, as eight of the ten flakes returning this score were at least 90 cm from the top of the gravel. We would stress that this observation is based on a small sample, and it is only through additional work that any taphonomic stratigraphic alignment can be determined.

Multiple authors stress there to be taphonomic variation within the known handaxe assemblage [2,4,20]. Ashmore [20] assigned abrasion based on a scale of one to five. From this, only four handaxes were regarded as perfectly fresh, 53 were a little worn, 151 were quite worn and 15 were heavily worn (although also see Roe [4]. Smith [2] noted one in 14 of the Fordwich handaxes to be heavily rolled. There is probably overlap between our ‘fresh to minimally worn category’ (*n* = 63, 25.1%) and Ashmore's [20] separate ‘fresh’ and ‘a little worn’ categories. Assuming this overlap to be correct, our flake data align well with these previous assessments and there is striking similarity in taphonomy between our flake assemblage and the known handaxes.

**Figure 8.**
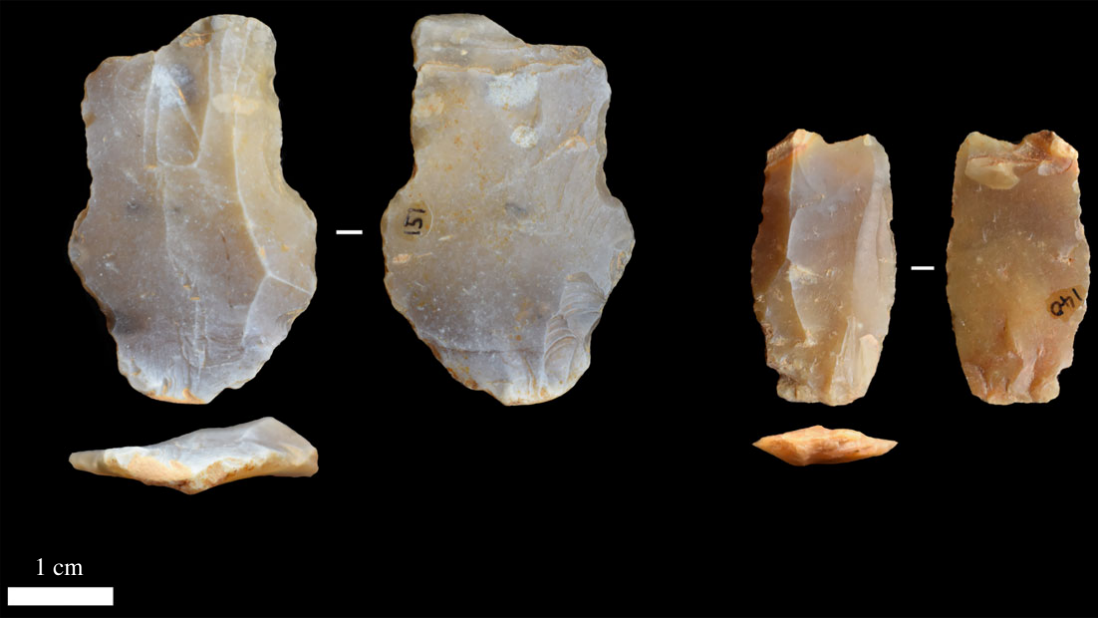
Flakes displaying features characteristic of potentially having been removed using soft-hammer percussion. The flake on the left was recovered from within the approximately 542 ka dated sand layer.

We have not recovered any handaxes to date and cannot contribute much to technological and morphological discussions on the existing Fordwich bifaces. However, contra Wymer [3], Roe [5] and Bridgland & White [18], we would not rule out the possibility of soft-hammer flake removals having been used to produce *some* handaxes at Fordwich. Indeed, while many flakes display cortex on their dorsal surfaces, few previous flake removals, and unprepared or minimally prepared flake platforms, and are therefore in agreement with previous studies identifying low numbers of removals on handaxes and the use of hard-hammer percussion [3,5,18], some do not. A few flakes display a prepared and small platform, diffuse bulbs of percussion, and low thickness to width ratios (figure 8), all characteristics associated with the soft-hammer technique [58]. Some display evidence of three or more previous flake removals on their dorsal surface, indicating heavier core-working than often assumed at Fordwich (potentially including heavily flaked handaxes, such as the example in figure 1). Most of the potential soft-hammer flakes were found in the upper main gravel (5 of 8 potential examples), so their presence during MIS 15 is dependent on the younger dates from this level being due to reworking. Potentially, some younger flakes (e.g. MIS 13) could have been reworked into these sediments during their MIS 10/11 disturbance. A sample of three from below the approximately 542 ka sand layer is not, in our opinion, large enough to conclusively argue for the presence of soft-hammer flaking.

## 6. Discussion

A century after Lower Palaeolithic artefacts were first discovered at Fordwich, the site is revealed to retain artefact-bearing gravels dating to approximately 542 000 years ago. These are among the oldest radiometrically dated artefact-bearing sediments in Britain and represent the fifth oldest archaeological occurrence currently known on the island (after Happisburgh, Fakenham Magna, Pakefield and Sapiston). Further, we provide evidence that the 330 handaxes recovered from Fordwich during the 1920s are older than approximately 437 ka, and are most plausibly contemporary with the newly excavated MIS 14 sediments. Assuming an absence of occupation during the cold MIS 14 glacial stage [10,34], Fordwich provides evidence of Acheulean hominins in southeast Britain during the MIS 15 interglacial (approx. 560–620 ka). This makes Fordwich the oldest directly dated Acheulean site in Britain. Fordwich is also revealed to be the only directly dated pre-MIS 13 Acheulean site in northern Europe to display a known handaxe assemblage numbering into the hundreds. After decades of only being mentioned in passing, Fordwich can now be considered a crucial piece of the pre-Anglian Palaeolithic puzzle in northwestern Europe.

No handaxes were discovered during these new excavations, but they have revealed technological components previously unknown at Fordwich. We provide the first evidence for the presence of scrapers, with Smith [2], Ashmore [20] and Roe [5] not previously reporting their presence. While limited in number, the scrapers are unmistakable, displaying direct continuous edge modification (figure 7). Two were collected from the base of the upper main gravel, while the scraper in figure 7 was found directly beneath the sand layer that dates to approximately 542 ka. We are hesitant to compare the Fordwich scrapers with well-documented examples from Warren Hill, High Lodge and Moulin Quignon [10,13], due to their limited number and fragmentary nature, but our initial impression is that they are smaller than those usually reported during the British Lower Palaeolithic and less intensively worked than those from High Lodge and Warren Hill. Whether this is derived from our selective sample, fluvial processes, or hominin intention, it remains to be seen. Additional retouched pieces were also found, including two notched flakes. It is, however, the 19 mm long double-pointed retouched implement that is most intriguing (figure 7). Seemingly retouched to create two points, it potentially represents the production of a very small piercing/boring tool. Assuming the younger MIS 10/11 age of the upper main gravel is the result of reworking, then its recovery from the base of these gravels may reflect an MIS 15 age for this artefact. A potential role for fluvial edge damage in the characterization of these implements cannot be ruled out entirely.

Similarly, we found evidence suggesting the production of cores smaller than those typically reported from the Lower Palaeolithic of northwest Europe. The example in electronic supplementary material, figure S3 appears to display eight removals from the same platform and was discovered *in situ* at the top of the lower main gravel (i.e. it is securely dated to approx. 542 ka). While it is easily differentiated from the chipped fluvial gravels at Fordwich, we remain hesitant in assigning it as a ‘miniaturized’ (cf. [59]) lithic artefact. A three-dimensional model of the object is available in the electronic supplementary material, and we invite critical appraisal of it. Three other cores are consistent with northern European Lower Palaeolithic reduction techniques (figure 7). The presence of small Lower Palaeolithic technologies should not be surprising, having been reported elsewhere in Britain (e.g. Beeches Pit, Layer 5a [22], and Boxgrove [unpublished]).

A large number of flake artefacts were discovered during the original quarrying work at Fordwich Pit [2], although little is known about their technological characterization. Our excavations discovered 238 flakes and flake fragments. These represent the total number to have been agreed on by all three analysts. While many are unmistakable (figures 6 and 8), and all contain multiple features indicative of being human made, we recognize that the fluvial deposition of these artefacts may mean a number (most likely smaller examples) could potentially be formed by non-anthropogenic processes. Smith [2] suggested that ‘Clactonian’ flakes, which we interpret to mean larger flake tools (in line with historical perceptions of these artefacts [60]) were previously found. Following Roe [5], we have not yet found any evidence of such tools.

Many flakes are consistent with having been detached during the manufacture of ‘crude’ minimally flaked handaxes (see above), as is generally suggested for the Fordwich bifaces [4–7]. Equally, some may represent expedient flake cutting tools produced through hard-hammer percussion on cores, despite their small size [61–63]. Their small size is not surprising as locally abundant fluvial flint nodules are generally less than 10 cm in maximum dimension. Some flakes display evidence of extended reduction sequences, with 40 exhibiting three or more previous flake removals on their dorsal surface. There is no depositional structure to the presence of these flakes, with broadly equal numbers in the upper and lower main gravel layers. Thus, we are confident that, at least on occasion, extended reduction sequences were being undertaken at Fordwich during MIS 15. Whether this was linked to more extended handaxe production sequences remains to be seen (see above discussion on soft-hammer flakes). There are no strong technological, morphological or taphonomic differences between flakes in the upper and lower main gravel, lending support to the notion all artefacts date from MIS 15 (table 5).

**Table 5.** Average technological, taphonomic and morphological comparisons between complete flakes in the intermediate coarse flint and upper main gravel (UMG), dated to MIS 10/11, and those from the sand layer and lower main gravel (LMG), dated to at least MIS 14.

	length (mm)	width (mm)	thickness (mm)	weight (g)	cortical coverage (%)	number of dorsal removals^a^	step, hinge and overshot terminations (%)	rounding scale
UMG	22.5	19.6	5.7	3.8	37.1	2.1	25	1.8
LMG	23.3	20.7	5.7	4.0	36.0	2.2	28.4	1.8

^a^of those displaying evidence of previous flake removals on their dorsal surface.

Prior to the recent discoveries at Fordwich, scrapers were unknown at the site [2,5]. Their presence below the approximately 542 ka sand layer confirms their MIS 15 production, making them among the oldest known scrapers in Britain. Confirming the MIS 15 age of the previously discovered handaxes is a little more complicated. Smith [2] notes the handaxes to have been recovered from ‘near the brow of the hill’, which would put them closer to our new MIS 14 excavations than the Bridgland *et al*. [7] exposures. Irrespective, at an absolute minimum the handaxes must date to approximately 437 ka as the main gravel mass at the 1998 exposure is below the sands dated to 423 ± 29 and 437 ± 29 ka. While we have not yet dated the lower main gravel at the Bridgland *et al*. [7] exposures, it is likely that, as with the lower main gravel at our recent excavations, they date to MIS 14 (electronic supplementary material, figure S2). Indeed, the Bridgland *et al*. [7] upper gravels and loam are only present in part of the western edge of the quarry (figure 3), while the lower main gravel stretches across the western edge of the Fordwich Pit between the 1998 and 2020 locations (figure 3). This covers the western edge of the pit near the brow of the hill, where most of the handaxes were recovered [2,4]. Our excavations reveal this sediment to contain artefacts dating to the early Acheulean period in Europe. Thus, at present, Fordwich's handaxes are most plausibly contemporary with our newly excavated MIS 14 sediments. The roughly shaped character of the Fordwich bifaces, which are atypical for MIS 12/13, further supports this interpretation [10,18].

### 6.1. Placing Fordwich among the wider Acheulean of Europe

Until now it has been difficult to place Fordwich among the wider Acheulean of Europe. The French site of Moulin Quignon displays evidence of Acheulean hominins in northwestern Europe during MIS 17 [13,34], and thus securely pre-dates Fordwich. From the five handaxes assigned to this early date, several are similar in form to those recovered at Fordwich (i.e. limited flake removals, relatively thick, and irregular in shape). Rampart Field (Suffolk, UK) has a single handaxe associated with an ESR date of 680 ± 26 ka, although it was recovered from greater than 1 m above the dated sediment and an MIS 15 age is preferred by the authors [32]. Warren Hill has a substantial collection of historically collected handaxes, with some argued to have been redeposited from MIS 15 contexts during the Anglian glaciation [10,32. Thus these artefacts could be direct contemporaries of those from Fordwich presenting a rare occurrence in the Pre-Anglian Lower Palaeolithic of northern Europe; two contemporary and substantial Acheulean assemblages. Brandon Fields and Maidscross Hill which both display large numbers of historically collected handaxes could also be from MIS 15 but are less secure in their attribution. Thus while they may be contemporaneous with Fordwich and display sizeable collections for comparison their precise place among the Acheulean of Europe is unclear. As it currently stands Fordwich is the only one of these four British occurrences with directly dated MIS 14 sediments demonstrated to contain artefacts. Thus Fordwich probably represents the joint second oldest Acheulean occurrence in northwestern Europe but it is currently the oldest after Moulin Quignon with radiometrically dated artefact-bearing sediments (see above regarding la Noira, Central France).

It is notable that the crude and highly rolled handaxes from Warren Hill conform with the elongated, thick, and poorly worked handaxes that characterize Fordwich [3–7,10,18]. Similarly, Brandon Fields and Maidscross Hill are also dominated by ‘cruder’ less heavily flaked handaxes, in line with those from Fordwich [10]. Together, these four sites seemingly indicate bifacial large cutting tool (LCT) technological uniformity in Britain during MIS 15 [16]. Currently, it is unclear how this similarity is related to cultural, raw material and reduction factors. The application of ‘quantitative genetic’ approaches will help to decipher the relative contribution of each of these factors [64]. Fordwich is the only MIS 15 context in Britain with confirmed evidence of scrapers. While further discoveries are required to fully explore their technological characterization, they appear closer to the MIS 17 Moulin Quignon scraper than the more highly worked MIS 13 examples from Warren Hill and High Lodge [10,13]. Their presence alongside handaxes further suggests that the MIS 13 separation in handaxe and scraper cultural groups, as suggested by Davis *et al.* [10], may not have an origin in MIS 15 populations.

The 700 000-year-old Acheulean site of la Noira in Central France pre-dates Fordwich by two MIS stages and is one of the most technologically diverse pre-MIS 13 sites in Europe, providing a sample of bifaces, flakes, cores and retouched pieces [65]. The technological similarity to Fordwich (at least as we currently understand it) is intriguing but, as stated above, a more detailed picture of the flake, core and retouched artefacts is needed in advance of any comparison. Similarly, once we have a more accurate understanding of the small artefacts from Fordwich, comparison with tools from Beeches Pit (MIS 11) could aid interpretation of the nature and extent of any technological continuity in small tool production through this period [22]. Comparison with small artefact assemblages recorded elsewhere in the European and the Levantine Lower Palaeolithic will identify similarities and differences at a continental level [59,66,67].

## 7. Conclusion

In 1981 ([5], p. 108) Derek Roe stated that ‘few Palaeolithic specialists outside Britain seem to have heard of Fordwich at all, though it could be one of the few substantial Early Acheulian sites of Europe’. Little changed in the subsequent 40 years. Through the above-described work, we hope that the importance of the site is now accepted. Fordwich reveals the presence of Acheulean hominins in what is now southeast Britain during MIS 15, a period dating to approximately 560 000–620 000 years ago. These populations produced minimally worked and elongated handaxes through hard-hammer percussion, although there are indications of more extended reduction sequences occurring. Combined with discoveries in the Bytham River and Somme valleys, it is now clear that hominins occupied large tracks of northwest Europe during MIS 15 and were almost certainly occupying a majority of what is now southern Britain (although this could be intermittently). While other demographic details remain elusive [16], the sizeable handaxe assemblages from Fordwich and the Bytham River may hint to prolonged occupations or populations sizes beyond a few explorative groups. Technologically, Fordwich is revealed to be more diverse than previously known, with handaxes, cores, flakes, scrapers and other retouched tools now evidenced. Several of the newly discovered artefacts appear to be unusually small, which is rare among the Lower Palaeolithic of Northern Europe, although the prevalence of such artefacts will only be revealed through further excavation. Together, Fordwich can now begin to take its place among the most important of early Acheulean sites in northwest Europe.

## Data Availability

All described artefacts are available through the University of Cambridge (no accession numbers yet recorded) or the British Museum (museum no. 19720901). The most important artefacts described here have been made available as three-dimensional models in the electronic supplementary material, Information [68]. IR-RF dating is destructive and therefore the samples used here are no longer available, but data were produced using the R-luminescence package v. 0.8.6. available at (https://CRAN.Rproject.org/package=Luminescence). All relevant IR-RF data is available in electronic supplementary material, Information table S1.
